# Adapting a palliative care intervention for people with advanced cancer across seven European countries: the Pal-Cycles intervention

**DOI:** 10.1186/s12904-026-02105-0

**Published:** 2026-06-12

**Authors:** Rachel Hooley, Sheila Payne, Holger Brunsch, Severine Marie Surges, Daniela Mosoiu, Flavia Hurducas, Pablo Hernández-Marrero, Sandra Martins Pereira, Ágnes Csikós, Éva Pozsgai, Wojciech Leppert, Maria Forycka-Ast, Pippa van den Brand, Jeroen Hasselaar, Nancy Preston

**Affiliations:** 1 International Observatory on End of Life Care, Division of Health Research, Faculty of Health and Medicine, Lancaster University, Lancaster, UK; 2 Department of Palliative Medicine, University Hospital Bonn, Bonn, Germany; 3 Medical Faculty, Transilvania University, Brasov, Romania; 4 Education and National Development Department, Hospice Casa Sperantei, Brasov, Romania; 5 Faculty of Sociology and Communication, Transilvania University, Brasov, Romania; 6 Universidade Católica Portuguesa, Católica Porto Business School, CEGE: Research Center in Management and Economics – Ethics and Sustainability Research Area, Porto, Portugal; 7 CEEAplA & Public Health Palliative Care Research Team, Universidade dos Açores, Fundação Gaspar Frutuoso, Ponta Delgada, Portugal; 8 Institute of Primary Health Care, University of Pécs Medical School, Pécs, Hungary; 9 Department of Public Health Medicine, University of Pécs Medical School, Pécs, Hungary; 10 Chair of Palliative Medicine, Institute of Medical Sciences, Collegium Medicum, University of Zielona Góra, Zielona Góra, Poland; 11 University Clinical Hospital in Poznań, Poznań, Poland; 12 Department of Primary and Community Care, Radboud University Medical Centre, Nijmegen, the Netherlands

**Keywords:** Healthcare, Palliative care, Healthcare systems, End of life, Advanced cancer, Cultural sensitivity, Patients, Family members, Cross-cultural, Stakeholder engagement

## Abstract

**Background:**

International adaptation of healthcare interventions requires sensitivity to local contexts, especially in palliative care, where healthcare systems and cultural expectations about end of life differ widely. Pal-Cycles is an intervention that aims to improve transitions in care for patients with advanced cancer. This intervention was adapted for implementation in a stepped wedge trial across seven European countries (Germany, Hungary, the Netherlands, Poland, Portugal, Romania and the UK). This paper aims to illustrate the process of adapting a palliative care intervention (Pal-Cycles) to meet the needs of those using healthcare settings across seven European countries.

**Methods:**

Adapted nominal group techniques (a structured group method that supports idea generation, discussion, and prioritisation) were used, involving both in-country and cross-country adaptation meetings focused on the five key components of the original intervention design, to ensure cultural sensitivity and best fit All countries established a group of clinicians and all except two countries (Portugal and Hungary) involved groups of patients and families. The adaptation process occurred in a series of 5 meetings, which were mostly held online to accommodate participants’ schedules.

**Results:**

A total of 36 clinicians, 14 patients or family members, and 16 facilitators participated in the adaptation process over a four-month period. Structured guidance and iterative consultation meetings ensured that the final intervention was both standardised and adaptable to each country’s healthcare setting. We produced a standardised intervention manual based on a theory of change model, ensuring consistency across countries while allowing for contextual flexibility.

**Conclusions:**

This paper provides guidance for future cross-cultural adaptation of palliative care interventions, illustrating the value of detailed methodological planning, structured guidance, and multi-stakeholder engagement in the adaptation process.

**Trial registration:**

ClinicalTrials.gov NCT06259136, registered on 6 February 2024.

**Supplementary Information:**

The online version contains supplementary material available at 10.1186/s12904-026-02105-0.

## Introduction

Conducting international research in palliative care is inherently complex due to differences in healthcare systems, levels of palliative care integration and development, and funding mechanisms across countries. These factors necessitate careful consideration when adapting interventions to ensure cultural, clinical, and systemic relevance [1]. In order to be effective, interventions developed in a specific context need to be adapted to the contexts where they will be implemented. This needs to be done efficiently and with sufficient expertise, requiring a structured approach. Furthermore, primary care and specialist palliative care across Europe varies widely in structure and strength. In some countries (e.g. the Netherlands, UK), General Practitioners (GPs) act as gatekeepers and coordinators of care, while in others, primary care plays a more limited role, with hospital specialists leading patient management. Levels of collaboration with secondary care, the scope of responsibilities, and the involvement of non-physician roles differ substantially, shaping how interventions like Pal-Cycles must be adapted [2].

Interventions developed for one population may not be effective when directly applied to other cultures or populations, while developing a new intervention for each population would be costly and time consuming [3]. Cross cultural adaptation is one approach to address this, which involves considering language, culture, and context in a way that it is compatible with the area’s cultural contexts, meanings, and values [4]. However, this approach has many challenges, including what has been described as the “Fidelity-Adaptation Dilemma” [5], which describes the tension between delivery of evidence-based interventions as developed to ensure effectiveness, and the need to address the needs of the local population by making changes to the intervention. Cultural adaptation considers where there is an area of intervention-population gap and makes changes to increase the relevance and fit [6]. However, this presents a risk that the intervention may be adapted in a way that decreases its effectiveness [3]. It has been suggested that fidelity can be maintained by strategic cultural adaptation that retains an intervention’s core theoretical components while making changes to ensure cultural relevance for the new population [5].

**Fig. 1:**
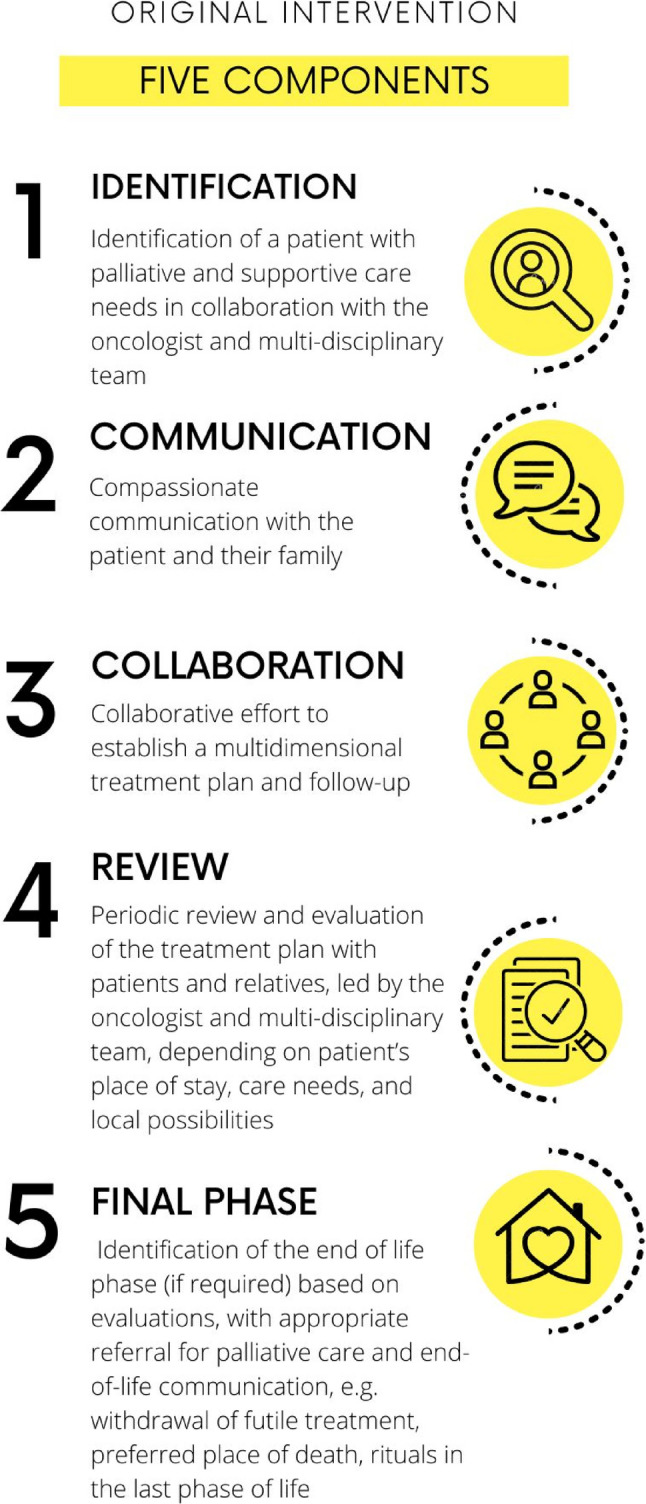
Original intervention

It has been identified that there is a requirement for high-quality evidence-based interventions for people with cancer with palliative care needs and their family caregivers [6, 7]. The Pal-Cycles intervention was originally developed in the Netherlands as part of a regionally integrated palliative care programme designed to improve coordination and continuity of care in the home setting with specialist palliative care backup from the hospital. The programme combined education for homecare nurses, structured collaboration between patients, families, primary care and specialist teams, and a shared palliative care protocol to support early identification and proactive planning. Implementation of this model was associated with a significant increase in home deaths, reduced hospital use and emergency visits, and lower healthcare costs during the last three months of life, demonstrating its impact on both care outcomes and system efficiency [8]. The intervention was based on five key components, previously described in a scoping review [8]: identification of palliative care needs, compassionate communication, collaborative treatment planning, regular review and evaluation, and recognition of the end-of-life phase (Fig. 1).

The original intervention, based on the five components, was adapted for the Pal-Cycles clinical trial [9], specifically for patients with advanced cancer in a hospital setting. The clinical trial involves testing a transitional care intervention between hospital and the community for patients with advanced cancer, through facilitating patient-centred communication and continuity of care. The trial aims to reduce unplanned hospital admissions and improve quality of life at the end of life.

The Pal-Cycles intervention, with its core components (Fig. 1), was developed and adapted considering context-based integration in (a) the local health system, (b) interorganisational collaborations (hospital-homecare), for implementation in (c) care practices for advanced cancer patients.

The aim of the paper is to illustrate the process of adapting a palliative care intervention (Pal-Cycles) to meet the requirements of healthcare settings across seven European countries. This process was guided by the ADAPT framework [10] and an adapted nominal group technique (NGT), a structured group method designed to encourage balanced participation, generate ideas, and enable collective prioritisation. Our adaptation modified the approach to enable cross-country participation and integration of patient and public involvement.

## Methodology

In order to adapt the Pal-Cycles intervention to each country’s local context, an adapted nominal group technique (NGT) was used to facilitate balanced participation across a series of consultation meetings, to encourage diverse viewpoints, and support consensus-building, ensuring that the intervention would be relevant and feasible across different clinical and cultural contexts [11]. The NGT provides a structured method to obtain information and ideas related to the area of interest, through encouraging inclusive balanced decision making [11, 12], without the need to achieve consensus across the group [12]. Typically, NGT are performed face to face and start with individual generation of ideas, which are ranked and prioritised through voting during the groups’ discussions although conducting online is known to be feasible [13]. As the current context involved adapting an intervention across several countries, an adapted NGT method was used.

The adaptation process used is described below. This adapted NGT method involved structured group meetings with multiple rounds of feedback and discussions. This iterative consultation process included both cross-country and country-specific meetings [11]. To guide the adaptation process, the ADAPT guidance [10] was used to ensure a systematic, evidence-based approach to tailoring the Pal-Cycles intervention across diverse healthcare contexts. The ADAPT framework emphasises key steps such as identifying core intervention components, assessing the need for adaptations, and systematically documenting changes to maintain fidelity while allowing for contextual flexibility. By following this guidance, the adaptation process ensured that the intervention’s core components, described above, remained consistent across countries. Simultaneously, the framework supported the integration of culturally and contextually relevant changes, such as modifying terminology or adjusting tools to align with local healthcare systems. This structured approach facilitated transparency, consistency, and rigor throughout the adaptation process, ensuring the intervention’s relevance and feasibility across all participating countries.

### Study design and setting

The adaptation process for the Pal-Cycles intervention spanned seven European countries: Germany, Hungary, the Netherlands, Poland, Portugal, Romania, and the United Kingdom.

### Recruitment of clinicians

Each country convened a group of clinicians, who were recruited purposefully through the professional networks of the research teams, to discuss the intervention’s components. Through purposive sampling we ensured diversity of roles (oncology, palliative care, nursing, primary care), care settings (hospital and community), and clinical experience with advanced cancer. Each adaptation meeting was planned to include 4–10 participants, balancing diversity of perspectives with the need for effective discussion. To account for potential non-attendance, additional participants were invited where feasible, and meetings were rescheduled or repeated in cases of significant absence. As a result, group sizes varied slightly between countries, but all meetings included adequate representation across key professional roles.

### Recruitment of patients and family carers

As cultural adaptation involves an iterative, and collaborative process, this should, where possible, include participation of those from the target population, for whom the adaptation is being developed [6]. Although this is still an emerging concept in some countries, five of the countries (Germany, the Netherlands, Poland, Romania and the UK) were able to convene a group of patients and/ or family members to engage in the adaptation process. These patients/ family members were recruited through the existing professional and community networks of the research teams in each country, including hospices, cancer support organisations, and clinical contacts. These Patient and Public Involvement (PPI) groups were invited to provide feedback on specific intervention components such as compassionate communication and the collaborative development of treatment plans.

To maintain consistency, the Lancaster University team (RH, SP, NP) developed standardised guidance, including a set of slides and instructions, to guide discussions (see appendix 1. For example). They also set up an initial cross-country meeting with the adaptation facilitators from each country and leading clinicians. This allowed generation of initial themes, to be later discussed in the first round of adaptation meetings in each country. This meeting also served as a workshop to demonstrate how to conduct the adaptation meetings effectively, to allow all participants to have equitable input on the discussion using the principles of NGT. Facilitators were members of the project team from each country (all with experience in qualitative methods and palliative care research) and were trained to ensure equitable participation, encourage contributions from quieter participants, and manage potential imbalances arising from professional hierarchies. Training included a demonstration session led by the Lancaster University team to standardise facilitation approaches and ensure consistency in how meetings were conducted, recorded, and documented across all countries. Agreement within groups was usually reached through clarification and discussion until a shared view was identified. Where differences remained, these were documented and fed forward into subsequent cross-country meetings, ensuring that divergent perspectives were acknowledged and considered in the final adaptation. A pragmatic approach to translation and documentation was used: facilitators summarised discussions in English using standardised templates. As facilitators were involved in the project this ensured conceptual understanding.

### Informed consent

Prior to participation, all individuals involved in the study; healthcare clinicians, patients, and family members, were provided with detailed written information outlining the purpose of the consultation meetings, and their rights as participants. Informed consent was obtained in writing from each participant, with signed consent forms collected and securely stored. Participants were assured of their right to withdraw from the consultation process at any time without consequence. This process was conducted in accordance with Lancaster University’s ethical guidelines and approval protocols.

### Stepwise adaptation process

The adaptation process occurred in a series of meetings, which were mostly held online to accommodate participants’ schedules and also to allow for sequential cross-country adaptation meetings to facilitate feedback. Only one clinician meeting and two patient and family meetings were held in person (see Table 1). The Lancaster team organised the process into the following stages, detailed below:

A

- ◦ Initial cross-country meeting (February 2023)
  - This initial meeting involved facilitators from each country, who were part of the research team on the Pal-Cycles project. An NGT facilitation style was demonstrated throughout the meeting as an example of how to effectively hold the adaptation meetings in each country. The meeting generated the key discussion points for the following adaptation meetings, which were structured around the five components identified in the original intervention design. Following this meeting the Lancaster University team wrote structured guidance in the form of PowerPoint slides, a guidance document and a feedback document, to ensure the key questions were asked in a similar manner and discussion points recorded consistently in each country.
- ◦ First round of adaptation meetings (March 2023)
  - The first adaptation round took place in March 2023. Clinician meetings were held in each participating country, while additional patient and family meetings occurred in Germany, the Netherlands, Poland, Romania, and the UK. The power point slides were translated as required. The guidance documents were used, with country teams summarising discussion points and submitting them to Lancaster for analysis. These meetings explored the relevance of each component, with participants identifying potential barriers and enablers in their healthcare context.
- ◦ Cross country meeting (April 2023)
  - A second cross country meeting with facilitators and some clinicians from each country involved the Lancaster University team presenting analysis from the first round of adaptation meetings, including areas of consensus and disagreement. These were discussed and key areas to be followed up in the second round of adaptation meetings created. Following this, the Lancaster University team created updated PowerPoint slides and guidance documents to be used in the second round.
- ◦ Second Round of adaptation meetings (May 2023)
  - The second round of adaptation meetings addressed the unresolved questions. Discussions focused on operationalising components, for example, who would be responsible for initiating conversations about care planning, whether patient-held forms should be digital or paper-based, and how ‘community teams’ should be defined. This round again involved clinicians from each country and the separate patient and family meetings held in Germany, Poland, Romania, and the UK, focusing on any elements of the intervention that required further consensus or contextual refinement [14].
- ◦ Final Cross-Country meeting (June 2023)
  - The last cross-country meeting served to finalise the intervention, integrating feedback from both rounds and addressing outstanding issues. This final round compared areas of agreement, divergence, where training could be helpful and implementation strategies. A draft intervention manual was then produced, which was shared with country team facilitators for final input and feedback. A final standardised version of the manual was the final result of the whole process.

Fidelity to the intervention was safeguarded by ensuring that the five core components (identification of needs, compassionate communication, collaborative treatment planning, regular review, and recognition of the end-of-life phase) remained unchanged across all countries. Decisions about which elements could be adapted were guided by the ADAPT framework and agreed collectively by the Lancaster team and country facilitators during cross-country meetings Adaptation was permitted in delivery mechanisms (e.g., role allocation, tool format, training models, terminology), but only where these changes supported feasibility and cultural relevance without altering the theoretical basis or intended outcomes of the intervention.

**Fig. 2:**
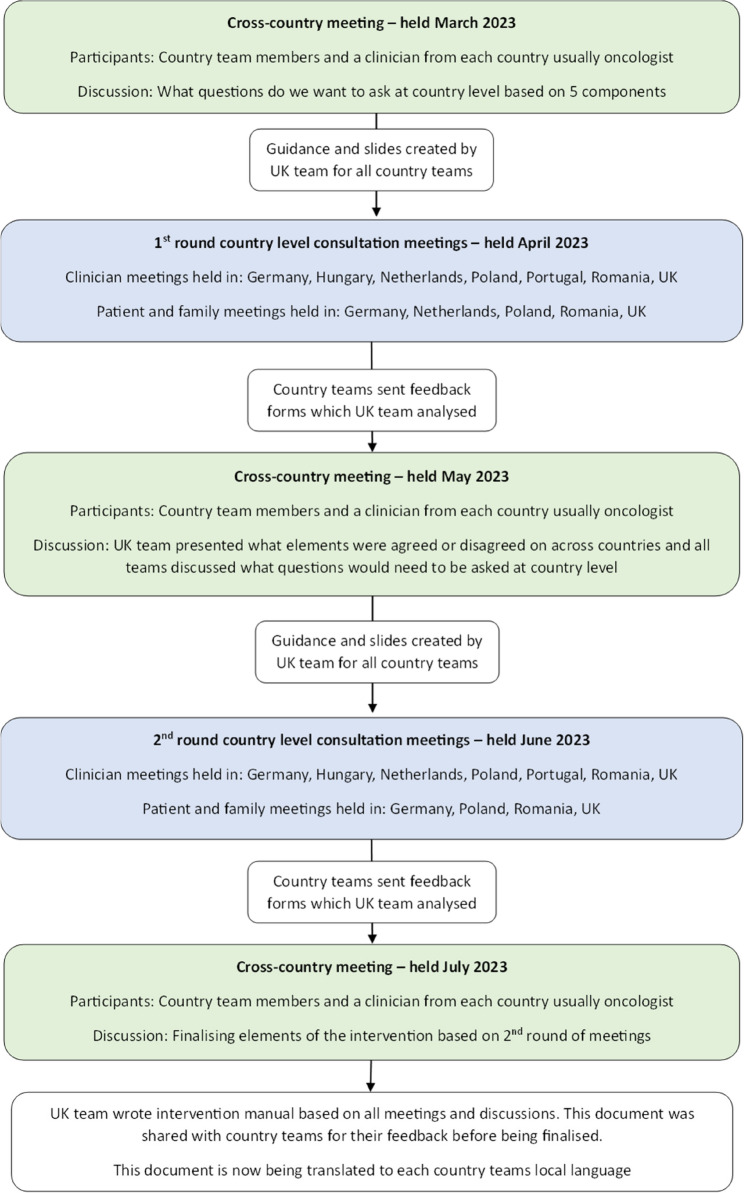
Process of adapting intervention

Meetings were conducted in the local language of each country. Rather than audio-recording, facilitators summarised discussions in real time using structured feedback forms, which were provided in English to ensure comparability across sites. Facilitators were fluent in both the local language and English, and the structured templates minimised the risk of loss of meaning during translation. The Lancaster team collated these forms and conducted content analysis, reviewing the summaries to identify key points raised under each of the five intervention components, areas of agreement and difference between countries, and any issues requiring clarification in subsequent meetings. The analysis was descriptive and comparative rather than coded, focusing on summarising and synthesising the main discussion points across countries to inform the next round of adaptation meetings. See Fig. 2 below showing the adaptation process.

We maintained detailed logs of the adaptation process, including changes made between rounds and the rationale for these changes. Cross-country meetings provided opportunities for reflection on both the content and the process of adaptation, allowing facilitators to share experiences of group dynamics and challenges in implementation. This iterative approach ensured transparency, supported learning across countries, and enabled us to document how decisions were reached and carried forward.

The process focused on summarising key points under the five intervention components, while noting any new issues raised. These summaries were compared across countries to identify commonalities and divergences. To balance variation and integration, we retained country-specific details in the summaries, but also drew out cross-cutting themes to inform the next round of discussions. Where perspectives differed, these were recorded explicitly and carried forward to subsequent cross-country meetings for consideration. This iterative approach ensured that originality and detail of local perspectives were preserved, while also enabling integration of ideas across countries.

## Results

A total of 38 clinicians participated in the adaptation meetings, including 18 oncologists, 3 oncology residents, 5 palliative care nurses, 5 GPs, 4 oncology nurses, 3 palliative care physicians, and a surgeon. In addition, 15 patient and public representatives took part (6 patients and 9 family members/representatives). Meetings were supported by 12 trained facilitators across the seven countries, see Table 1 below. The structured guidance materials provided by the Lancaster team enabled country partners to conduct consistent and culturally responsive meetings. Feedback indicated that flexibility was crucial in several areas, particularly in selecting culturally appropriate palliative care assessment tools [15].

**Table 1:** Participants in clinician and patient/public involvement (PPI) adaptation meetings across seven countries

Country	Clinician participants (*n*, roles, gender)	PPI participants (*n*, roles/characteristics, gender if known)	Meeting format	Facilitators
Germany	2 clinicians: oncologists (dermatology, urology), both Female	3 participants: 1 patient, 1 family member, 1 patient rep (self-help group)	Online	2
Hungary	10 clinicians: 6 oncologists, 3 oncology residents, 1 surgeon; 10 Female/4 Male	No PPI group	Online	1
Netherlands	4 clinicians: 1 GP, 2 oncologists (palliative care), 1 oncology nurse; 2 Female /2 Male	1 family member	Clinicians: online; PPI: face-to-face	2
Poland	5 clinicians: 1 PC nurse, 1 oncologist, 1 oncologist/PC specialist, 1 PC/geriatrics specialist, 1 PC & family doctor; 2 Female/3 Male	4 participants: 1 patient, 2 family members, 1 nurse	Online	1
Portugal	8 clinicians: 2 oncologists, 1 GP, 5 nurses (specialist/generalist, PC and mental health); 6 Female/2 Male	No PPI group	Mixed (one in-person + one online)	2
Romania	5 clinicians: 2 oncologists, 1 GP, 1 PC physician, 1 PC nurse; 4 Female/1 Male	4 patients	Clinicians: online; PPI: face-to-face	2
UK	4 clinicians: 2 oncologists, 1 PC nurse, 1 GP; all Female	4 participants: 3 family members, 1 patient	Online	2

As part of the adaptation process, structured consultation groups, including facilitators from the Pal-Cycles research teams and healthcare clinicians, were established in each of the seven participating countries to tailor the intervention to local healthcare practices. In each country, a group of healthcare clinicians (including GPs, oncologists, palliative care physicians, cancer nurse specialists, and palliative care nurse specialists) was formed. This diversity in clinical expertise was integral to understanding the varied perspectives within multidisciplinary cancer and palliative care teams. By involving professionals from both primary (GPs) and specialist care (oncology and palliative care), the adaptation process benefited from insights into each stage of patient care, including initial diagnosis, ongoing treatment, and palliative support.

In five countries, meetings with patients and family members ensured that end-user perspectives were central to the adaptation process. Feedback from the patients and family member meetings was incorporated when adapting the intervention across all countries, such as patients’ request for more involvement in their own care plans, and the idea of a patient held form. The final intervention manual incorporated standardised elements but allowed flexibility for country-specific applications, such as defining “community teams” based on each healthcare context. The patient and family groups included current patients, relatives, bereaved relatives and patient representatives from self-help group associations.

Participants of the adaptation meetings highlighted both the opportunities and challenges of the proposed intervention components. Patient and family representatives valued tools such as symptom forms, noting that “being able to fill in the form myself feels empowering, but only if I know it will lead to something” (UK PPI participant). Others emphasised the importance of support when completing these tools, suggesting that “having someone you trust to go through it with you would be really helpful” (UK PPI participant).

Similar reflections were echoed across countries. In Germany, participants felt forms should also include psychological and social aspects but cautioned against making them too burdensome for patients. In Romania, patients suggested using visual scales or symbols to make symptom reporting easier and stressed that caregivers should not complete forms on their behalf, as “symptoms are subjective experiences.” Clinicians in Poland highlighted that digital versions (e.g., on tablets) might improve feasibility.

Across all settings, open and compassionate communication was viewed as essential. A German family member described good practice as “having several conversations where we felt recognised in our individuality,” contrasting with experiences of rushed, impersonal hospital communication. Romanian patients similarly stressed that doctors should “adapt the discussion to the patient’s emotions and capacity to understand.” Clinicians in Hungary and the Netherlands pointed out that while oncologists are confident communicators, structured discharge letters and clear role definitions could improve transitions between hospital and primary care.

Clinicians across countries also emphasised practical implementation challenges. Hungarian teams preferred in-person training with real case discussions, while Dutch oncologists felt further communication training was unnecessary but requested focused sessions on referral and transition to primary care. Polish clinicians noted the difficulty of holding in-depth care planning conversations during short appointments but agreed that structured tools could help.

These examples demonstrate how the intervention needed to remain flexible to reflect national differences in healthcare systems, communication practices, and professional training while retaining its core principles.

All adaptation meetings, including the guidance documents were based on the key components of the original intervention design. However, within these key areas there needed to be flexibility in definitions and implementation across countries. Therefore, part of the adaptation process was related to which areas could be flexible across contexts and which elements of the intervention had to be defined and implemented the same in all countries to maintain the key components.

During the development of the intervention, key elements were established to ensure consistency across all countries. For instance, the Edmonton Symptom Assessment System Revised (ESAS-r) was chosen as the symptom assessment tool. While ESAS-r is commonly used in European countries, it is not typically used in the UK, however, maintaining a standardised tool across all countries was essential.

At the same time, certain aspects of the intervention design required flexibility to adapt to local contexts. For instance, responsibility for initiating conversations about care planning differed, in some countries oncologists typically led these discussions, while in others they were undertaken by specialist nurses or GPs. Patient-held forms and questionnaires used during the intervention remained the same across all countries, however the way in which they were administered varied, with some countries using online versions and others using paper format. Similarly, as the current intervention is based on transitions which may include transitions from hospital into the community, transitions in goals of care or transitions in care management [16], there was flexibility in defining terms used in the intervention, such as “community teams,” which varied based on the structure and context of each country’s healthcare system. In addition, differences emerged in how training for clinicians was organised. All countries required training to support implementation of the intervention. However, the background training related to cancer care and palliative care varied in scope and intensity according to local needs and existing education systems. In some countries, where background education in cancer and palliative care was already widely and freely available (for example, in the UK and the Netherlands), shorter in-person or online “refresher” sessions were considered sufficient to update clinicians on the specific elements of the Pal-Cycles intervention. In contrast, in countries where such specialist training was less readily available (for example, Romania and Hungary), more extensive, foundational training sessions were regarded as essential to ensure a shared understanding of palliative care principles and intervention delivery. These decisions reflected what was feasible and realistic for clinicians in each setting, ensuring that training was appropriate to local practices and maximised participation.

These examples illustrate the diversity of perspectives and alternative approaches that emerged across countries. At times, disagreements arose, for instance, over who should lead conversations or which training models were most feasible. Such differences were carried forward to cross-country meetings, where they were either resolved by agreeing on a flexible approach (e.g., allowing country-level choice of lead clinician) or retained as acceptable contextual adaptations. This process ensured fidelity to the five core components while accommodating local variation. Clinicians tended to contribute more regarding feasibility and workflow, while patients and family members highlighted usability and communication. This iterative process ensured that both areas of agreement and contention informed the final intervention design.

The series of meetings also played an important role in shaping the final intervention. The iterative process allowed initial ideas and challenges to be raised in national groups, tested and compared at cross-country level, and then refined in subsequent rounds. For example, unresolved issues from Round 1 (such as role allocation and training models) were explicitly carried into Round 2 for further discussion, while the final cross-country meeting ensured that remaining differences were reconciled and incorporated into the intervention manual. Having five rounds was therefore not redundant but necessary to provide opportunities for escalation, reflection, and refinement. This structure ensured that the final intervention balanced consistency across countries with appropriate local flexibility.

**Fig. 3:**
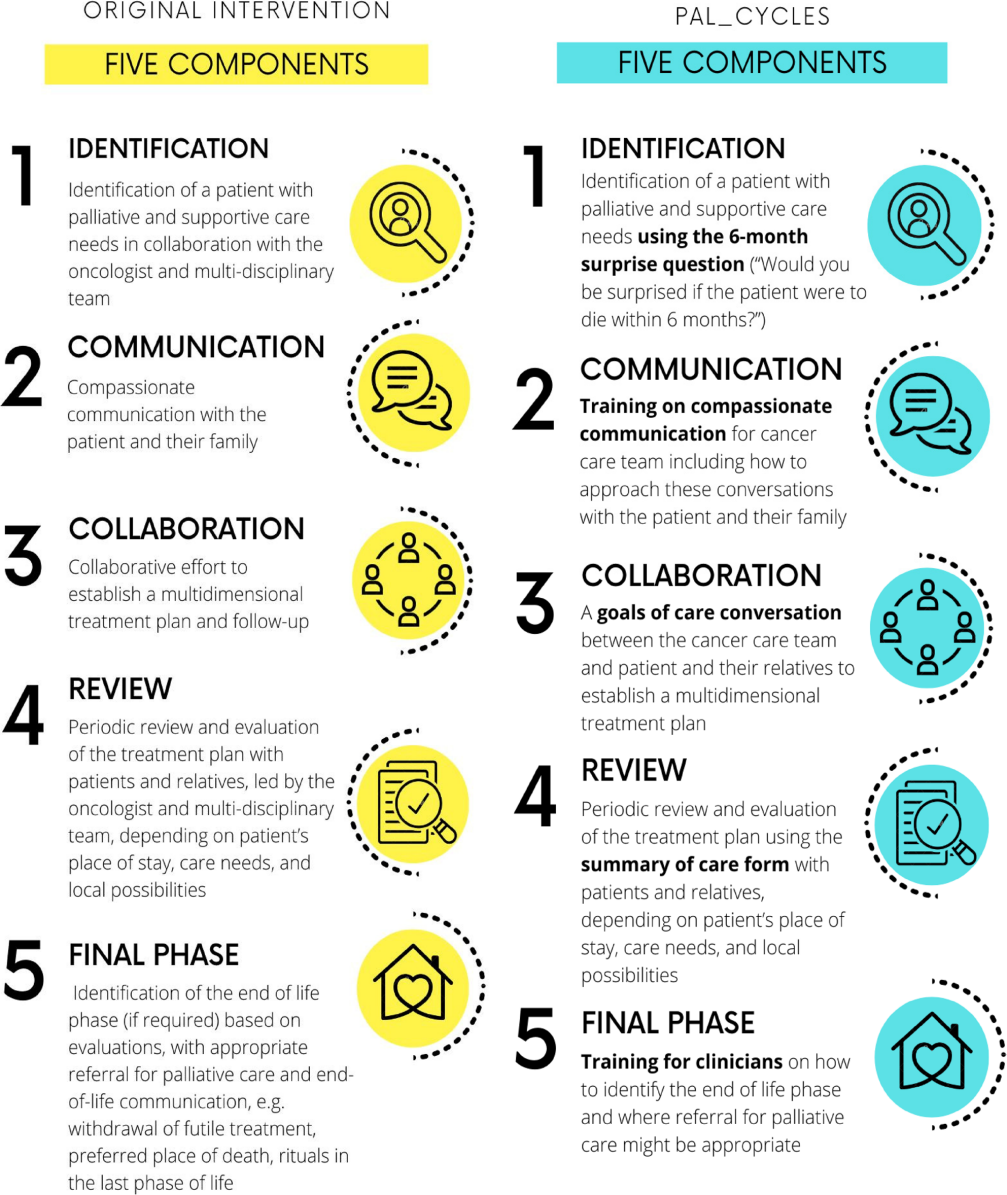
Original intervention components implemented for Pal-Cycles

As a result of the adaptation meetings, an intervention manual was developed, including the adapted intervention design. This adapted intervention design for Pal-Cycles can be seen below (Fig. 3), which shows how the 5 elements from the original design will be implemented. The intervention manual was then translated into local languages of the seven partner countries, where the intervention will be trialled.

**Fig. 4:**
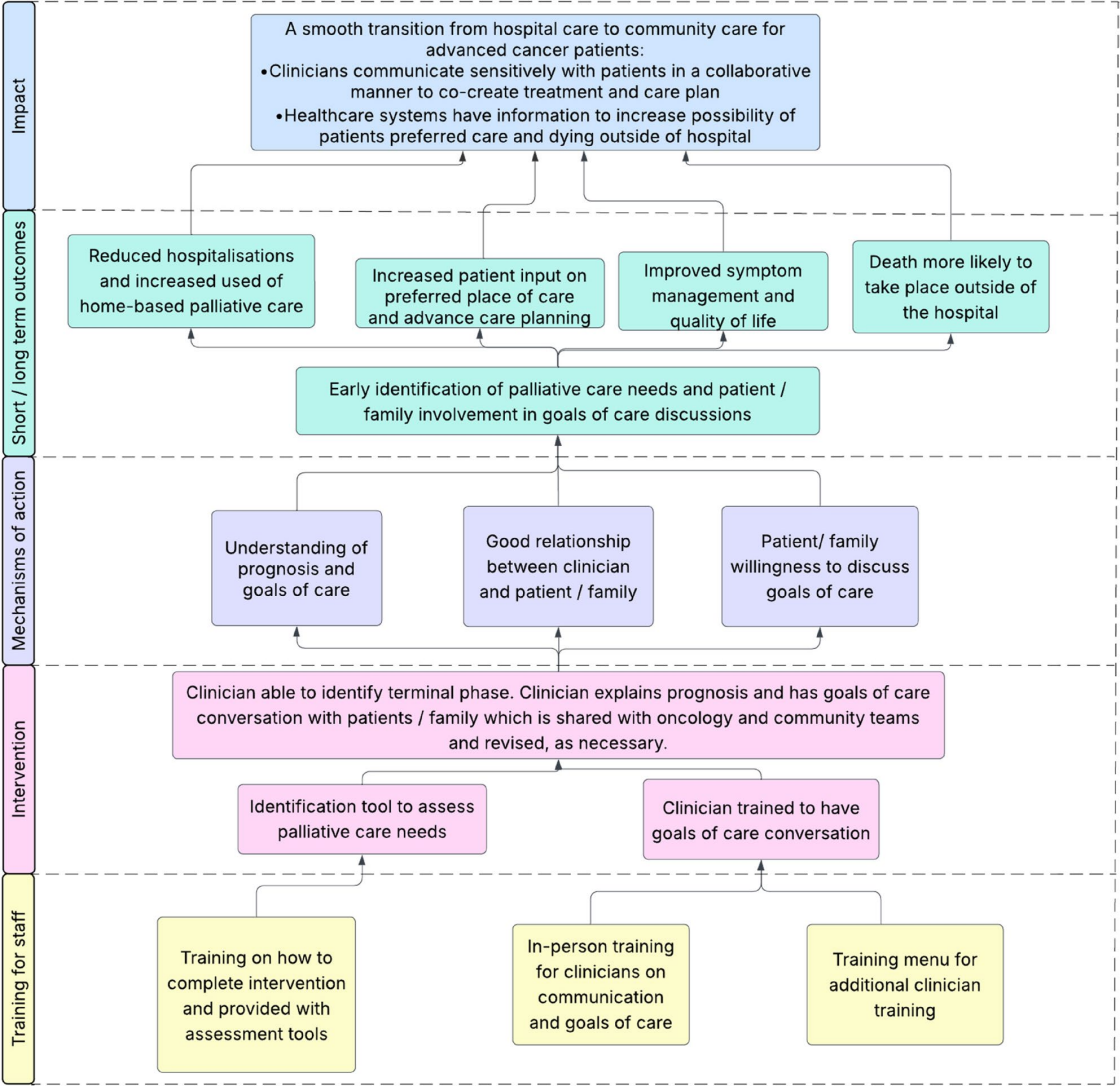
Theory of change model for the Pal-Cycles intervention

As a result of the adaptation meetings, it was important to consider the underpinning elements and mechanisms necessary for the intervention to have its desired impact. Therefore, a theory of change model was also created to help us understand how the Pal-Cycles intervention may affect the transition process and care trajectory of cancer patients. The theory of change model considered the following dimensions, and is shown below (Fig. 4):

- The goal of the intervention
- Long term outcomes
- The preconditions that need to exist for impact to be achieved
- The ceiling of accountability at which we would stop measuring if outcomes have been achieved
- Indicators that can measure progress achieved in relation to outcomes
- The intervention strategies
- Rationale
- Assumptions

## Discussion

This paper demonstrates the feasibility and advantages of a structured, iterative process for international intervention adaptation. By involving stakeholders from multiple countries, the adaptation process ensured that diverse perspectives informed the intervention’s design, leading to a final product that was both standardised and locally adaptable [17]. Using adapted NGT allowed the clinicians to systematically discuss and prioritise adaptation needs, identify barriers to implementation, and suggest practical adjustments suited to their local health systems.

The adapted version of NGT used differs from traditional NGT to suit adaptation of an intervention for seven European countries. For example, NGT is usually done using face to face methods, however, to ensure feasibility for clinicians working busy schedules, and to allow countries to include international participants where necessary, our adaptation meetings were held online, with only two patient and family meetings held in person. Furthermore, typically NGT uses ranking of priorities through voting and result in both qualitative analysis (analysis of recordings) and quantitative analysis (numerical ranking of ideas) [13]. In this case, as we developed the intervention between seven different countries, the aim was not to identify a single solution, but to assess how the key components of the intervention could be implemented across all contexts. Rather than using the traditional ranking method, the cross-country meetings enabled feedback from all countries to be considered, before confirming or re-generating ideas suitable for all as part of an iterative process.

The cross-country meetings considered cultural sensitivity and best fit, allowing ideas to be generated regarding how elements of the emerging intervention may be defined differently in each setting. Reliability of this cultural adaptation process was achieved through focussing on the five core theoretical components of the original intervention design throughout discussions which were maintained [5].

Across five of the seven participating countries (Germany, the Netherlands, Poland, Romania and the UK), Patient and Public Involvement (PPI) was also established. PPI groups are increasingly recognised as essential to the development and adaptation of healthcare interventions, ensuring that the patient perspective is integrated into research and practice [18]. The PPI groups consisted of patients and family members, who provided insights based on their experiences, bridging the gap between research and patient-centred care [19]. Engaging PPI groups is especially valuable when developing palliative care interventions, where understandings of patient needs and preferences are critical. Research has shown that PPI involvement can improve the relevance, quality, and feasibility of interventions by grounding them in the priorities of those directly impacted by healthcare services [20]. For cross-country studies like the adaptation of the Pal-Cycles intervention, PPI groups also offer contextual insights that ensure the intervention aligns with cultural factors within each country. For example, in the current study the PPI groups highlighted a need for improved and more compassionate communication, as well as suggesting the use a patient held form to improve communication regarding treatment and care plans. These elements were incorporated into the final intervention design.

PPI engagement is increasingly recognised as a cornerstone in designing healthcare interventions, as it bridges the gap between clinical objectives and patient priorities [19]. While PPI engagement is still an emerging concept in some of the participant countries, the inclusion of patients and family members in five countries added valuable insights, highlighting the importance of user-centred approaches in intervention development. Their contributions helped refine the intervention, ensuring that it meets the needs of patients and families facing advanced cancer and palliative care across diverse healthcare settings. Furthermore, the structured guidance supported consistency across countries, emphasising the need for detailed, adaptable frameworks in international research collaborations [21]. The absence of PPI groups in two countries (Portugal and Hungary) may have limited the depth of cultural insights obtained. While experienced clinicians contributed valuable perspectives, future research should prioritise developing feasible models for PPI engagement in contexts where this remains an emerging practice, to strengthen cultural and contextual adaptation. Overall, the adaptation process demonstrated that different countries and professional groups contributed distinct insights shaped by their healthcare structures and roles, while patient and family members emphasised usability and communication. Comparing countries with and without PPI also highlighted the added value of incorporating patient perspectives, which drew attention to priorities that might otherwise have been overlooked.

Despite the usefulness of using NGT, it could allow inadvertent bias towards more dominant voices within groups or hinder deeper exploration of opposing perspectives [11]. Therefore, ensuring balanced participation, especially in settings which could be perceived to be hierarchical, requires careful facilitation to avoid biased discussions [21]. This was facilitated through frequent turn taking when discussing ideas throughout the meetings.

Furthermore, while it is important to involve PPI groups in this type of research, it is also important to ensure the representativeness of the PPI groups. As PPI groups were established in only five countries, there may have been some unique cultural insights that were missed from the other two countries. Given the cultural variability in perceptions of palliative care and death, broader PPI engagement could have further enhanced the intervention’s contextualisation [22].

The flexibility incorporated into the Pal-Cycles intervention, such as tailoring assessment tools and defining “community teams,” enhances the ability to implement the Pal-Cycles intervention across various European countries. However, it is important to ensure consistency in implementation, through maintaining core intervention components while allowing contextual modifications. This can be a challenge in healthcare interventions, as excessive flexibility could weaken results [15].

The model presented, involved using adapted NGT including multiple rounds of adaptation meetings, with multiple groups of key stakeholders and cross-country meetings with facilitators. The use of structured guidance was key to ensure consistency and ensuring that intervention elements remain consistent with key components of the original intervention across all countries increases the fidelity of the cultural adaptation of the intervention. This was a valuable method for international adaptation of a healthcare intervention. It emphasises the balance between standardisation and local adaptation. The iterative consultation process aligns with best practices in developing complex interventions, which advocate for systematic stakeholder engagement and phased development [17].

## Conclusion

Adapting a palliative care intervention across multiple countries requires structured, systematic approaches to facilitate consensus while respecting cultural and systemic differences. The adapted nominal group technique, combined with structured guidance, enabled successful adaptation of the Pal-Cycles intervention across seven European countries. The process resulted in a standardised intervention manual, with flexibility for country-specific (cultural, care setting specific) adaptations, underscoring the importance of adaptable, structured methods in the development of international healthcare interventions [23]. This study provides a model for future cross-cultural adaptation of palliative care interventions, illustrating the value of detailed methodological planning, structured guidance, and multi-stakeholder engagement in the adaptation process.

## Supplementary Information

Supplementary Material 1.

## Data Availability

No datasets were generated or analysed during the current study.
