# Supplementary material for: Adapting a palliative care intervention for people with advanced cancer across seven European countries: the Pal-Cycles intervention
Source: BMC Palliat Care. 2026 Jun 12;25:181. doi: 10.1186/s12904-026-02105-0 (PMC13289543; doi:10.1186/s12904-026-02105-0)
Supplement: Supplementary file 1 — Supplementary Material 1. [file 12904_2026_2105_MOESM1_ESM.pdf]

Appendix 1. Guidance materials example – round 1 document and slides provided to country teams to guide PPI meetings

Pal-Cycles WP1 Guidance on how to organise and conduct consultation meetings with patients and families

This document offers guidance on how to organise and conduct WP1 consultation meetings with patients and families (or their representatives) who you have identified to be part of your 'country teams'. Please use this information in addition to the Protocol for WP1. We encourage you to use it flexibly in line with your country requirements. Text in *italics* is designed to provide additional direction to help you. Key steps are in **Red**. We have prepared some Power Point slides that you might need to adapt to your language and country context.

Step 1 – Arrange meetings: *You will need to do one consultation meeting with patients and families in March and another one in May (ideally with the same people)*

- Select people to join your country team, these can include (minimum of 3 participants from any group):
  Patients, families, bereaved relatives, patient representatives.

- Agree meeting dates / start time *(We suggest that you allow 60-90 minutes for the meeting)*

- Agree on venue for meeting either face-to-face / online.

Step 2 – Before the meeting:

In advance of the first meeting send out:

1. Participant information sheet
2. Consent form (if you submitted an ethics application and have approval)

Step 3 – Guidance on conducting the consultation meeting No1 with patients/ families:

Introductions: Introduce yourself and your research team members. Explain the purpose of the meeting and the overall aims of the project. Then go around the consultation meeting participants inviting them to briefly introduce themselves to the group – suggest that they use their first name only. Remind participants to be respectful of everyone's views. Check that they understand the words 'cancer' and 'palliative care'. Warn everyone that the meeting will cover some sensitive topics that might be distressing to talk about.

Introduce to the consultation meeting:

(Slide 2)

Introduce the Pal_Cyles project and aims:

(Slide 3)

First component: Identification

Doctors and nurses need to be able to recognise when a person has palliative care needs, in a timely manner to ensure that they have appropriate care.

Questions for the group:

- How did your doctor or nurse recognise that you or your family member might have palliative care needs?
- How did process feel like for you (or your family member)?

Note down patient / families experiences

(Slide 4)

Second component: Compassionate communication

In our project, we want to help doctors and nurses communicate in ways that are compassionate and sensitive. This will involve an open conversation about a patient's 'goals of care' and 'advance care planning'. Check that participants in the group understand these terms, if not, offer definitions that are appropriate in your country.

Questions for the group:

- What are your experiences of communication with doctors and nurses about your cancer or your family member's cancer?
- Were there any instances when communication with you/ your family could have been improved?
- If so, how could this be improved?

Note down patient / families experiences

(Slide 5)

Third component: Collaborative effort to establish treatment plan

Doctors and nurses need to work together to develop a treatment plan to help cancer patients. They often need to communicate this treatment plan to other doctors and nurses working elsewhere or in other teams. Doctors and nurses may also talk with patients and families in developing treatment plans. Sometimes these treatment plans are called 'goals of care' conversations as they make plans for future care options.

Questions for the group:

Note down patient / families experiences

In our project, we want to keep a record of the 'goals of care' conversations doctors and nurses have with patients and families and how they share decisions about any future treatment plans.

- Have you ever had a conversation with a doctor or nurses about your treatment plan?
- Have you had any meetings with doctors and nurses about your/ their treatment plan?
- If so, were they useful?
- Who was involved in these conversations?
- Where and when did these conversations occur?
- If there was no conversation, do you think this would be useful? And when should this conversation happen?

(Slide 6)

Fourth component: Review and evaluation of treatment plan (Goals of care)

Overtime treatment plans for cancer often need to be reviewed to check that things are working well or need to be changed. Doctors and nurses may meet together to talk about and review treatment plans. In some places, patients and families join these meetings to share in the conversation.

Questions for the group:

- In your experience, are you aware of when (or if) your/their treatment plan was discussed at a meeting with doctors and nurses?
- Were you/ your family involved in this review/ evaluation of the treatment plan?
- Was the evaluation of the plan done at a set time? – If not, when do you think it would be helpful to do this evaluation?
- How often is it helpful to have these review meetings?

Note down patient / families experiences

(Slide 7)

Fifth component: Recognising when the patient becomes very ill and may not recover

Doctors and nurses need to be able to recognise when a person has become very ill and may not recover so they can help everyone prepare and to ensure that the right sort of care is provided.

Questions for the group:

- What is your experience of recognising that someone in your family has become very ill and may not recover?
- Would being able to talk about this be helpful for you and your family and make you feel better prepared?
- If not, is this something you think would be helpful to have written information about?
- If so, in what format/ how would you prefer to receive this information?

Note down patient / families experiences

(Slide 8)

Closing the consultation meeting

Thank everyone for sharing their experience. Remind people about confidentiality. Ask if anyone has any other questions.

Thank participants for their time and let them know you will be in touch regarding dates/ times for the next workshop (if not already agreed)

Ensure that if they are distressed by the consultation meeting that they can seek help from (identify local organisations here)

(Slide 9)

Prepared by Rachel Leech, Sheila Payne and Nancy Preston

Version 2, 7th March 2023

Guidance for questions to use at consultation meetings with patients/ families

Pal-Cycles Work Package 1
1st Consultation meeting – with patients/ families

Introduction to the consultation meeting

- Introduce everybody present
- The aim of the meeting is to share your (or your family's) experiences of cancer care
- There are no right or wrong answers
- We value everybody's experiences
- We do not expect you to disclose any personal information
- We ask that all experiences are not repeated outside of this meeting

International Observatory on End of Life Care | Lancaster University

Overview of our study

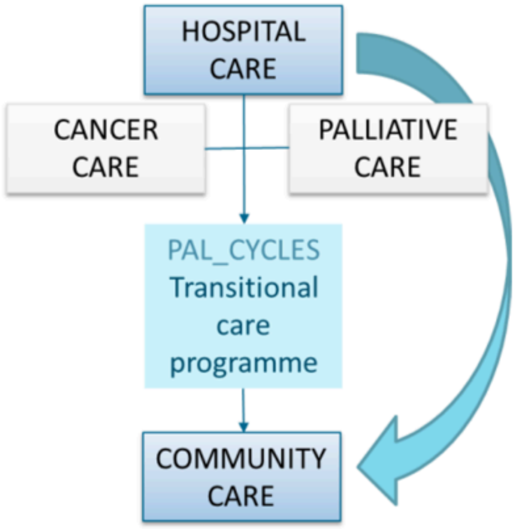

- Our study aims to improve transitions in care for people with cancer
- We will create a new plan of care which will be tested in 7 European countries

International Observatory on End of Life Care | Lancaster University

Identification

Doctors and nurses need to be able to recognise when a person has palliative care needs, in a timely manner to ensure that they have appropriate care.

- How did your doctor or nurse recognise that you or your family member might have palliative care needs?
- Did this work well for you?

International Observatory on End of Life Care | Lancaster University

Compassionate communication

- What are your experiences of communication with doctors and nurses about your cancer or your family member's cancer?
- Were there any instances when communication with you/ your family could have been improved?
- If so, how could this be improved?

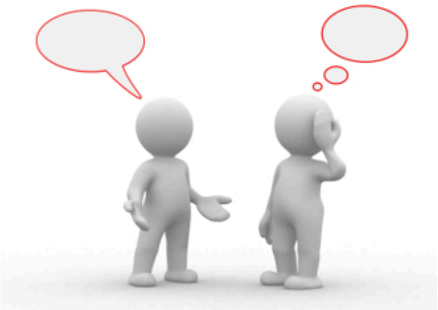

International Observatory on End of Life Care | Lancaster University

Collaborative effort to establish treatment plan

- Have you ever had a conversation with a doctor or nurses about your treatment plan?
- Have you had any meetings with doctors and nurses about your/ their treatment plan?
- If so, were they useful?
- Who was involved in these conversations?
- Where and when did these conversations occur?
- If there was no conversation, do you think this would be useful? And when should this conversation happen?

International Observatory on End of Life Care | Lancaster University

Review and evaluation of treatment plan

- In your experience, are you aware of when (or if) your/their treatment plan was discussed at a meeting with doctors and nurses?
- Were you/ your family involved in this review/ evaluation of the treatment plan?
- Was the evaluation of the plan done at a set time? – If not, when do you think it would be helpful to do this evaluation?
- How often is it helpful to have these review meetings?

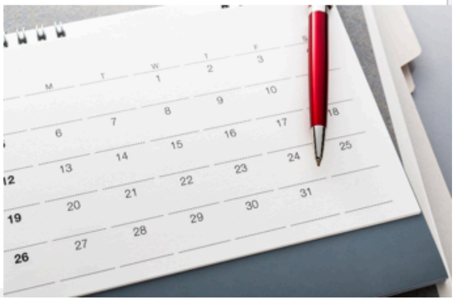

International Observatory on End of Life Care | Lancaster University

Recognising when the patient becomes very ill and may not recover

- What is your experience of recognising that someone in your family has become very ill and may not recover?
- Would being able to talk about this be helpful for you and your family and make you feel better prepared?
- If not, is this something you think would be helpful to have written information about?
- If so, in what format/ how would you prefer to receive this information?

End of meeting

- Thank you for sharing your experiences

- *If you wish to contact us about the project or the meeting today please contact (name of contact)*

- *If you would like support please contact (insert country support resources or contacts)*
